# DISABILITY STATUS OF ASIAN OLDER ADULTS IN THE UNITED STATES: FINDINGS FROM THE 2017–2021 AMERICAN COMMUNITY SURVEY

**DOI:** 10.1093/geroni/igae098.1789

**Published:** 2024-12-31

**Authors:** Christina Miyawaki, Hoang Nguyen, Kyriakos Markides

**Affiliations:** University of Houston, Houston, Texas, United States; The University of Texas Medical Branch, Galveston, Texas, United States; The University of Texas Medical Branch, Galveston, Texas, United States

## Abstract

Little information is available on the disability status of the diverse Asian population in the United States (U.S.). In this study we estimate the prevalence of six types of disability in older adults for six major Asian subgroups using the recently released data from the 2017-2021 American Community Survey. The survey contains a sample of 11,737 Vietnamese; 32,388 Chinese; 24,625 Filipino; 12,125 Japanese; 10,231 Koreans; 16,759 Indians; and 2,412,999 non-Hispanic Whites. All analyses use survey weights for population estimates and the jackknife method for standard errors. We find higher prevalence of disability in independent living for Vietnamese, Chinese, Filipino, Japanese, and Indian compared to non-Hispanic White. For self-care disability, Vietnamese, Chinese, Filipino and Indian have higher prevalence. Cognitive disability is more prevalent in Vietnamese, Filipino, and Japanese. Only Vietnamese older adults have higher prevalence of mobility disability and vision disability. Surprisingly, all Asian subgroups in the study have lower prevalence of hearing disability than the non-Hispanic White group. Overall, the results show Vietnamese older adults have the worst disability profile, whereas Korean older adults have the best profile. Future research is needed to understand the role of fortitude in addressing disability status, especially in the areas of independent living, self-care, and cognition, which are more prevalent in most Asian groups.

